# Supplementary material for: Perioperative changes in left ventricular systolic function following surgical revascularization
Source: PLoS One. 2022 Nov 10;17(11):e0277454. doi: 10.1371/journal.pone.0277454 (PMC9648779; doi:10.1371/journal.pone.0277454)
Supplement: S1 Table — (DOCX) [file pone.0277454.s001.docx]

***Supplemental Table 1. Odds Ratios of Battery of Potential Predictors for LVEF Improvement***

| **Variable** | **Odds Ratio** | **95% CI** | **p-value** |  |
| --- | --- | --- | --- | --- |
|  |  |  |  |  |
| **Preoperative EF** | 1.3136 | 1.17-1.48 | <0.001 |  |
| **SVR** | 1.6365 | 1.09-2.45 | 0.017 |  |
| **Digoxin** | 0.5885 | 0.32-1.08 | 0.09 |  |
| **Warfarin** | 1.559 | 0.86-2.82 | 0.14 |  |
| **Hypertension** | 0.8477 | 0.57-1.27 | 0.42 |  |
| **NYHA III/IV** | 1.4322 | 0.95-2.15 | 0.08 |  |
| **RN (vs. Echo)** | 1.5017 | 0.85-2.65 | 0.16 |  |
| **CMR (vs. Echo)** | 1.4224 | 0.91-2.20 | 0.13 |  |

Abbreviations: CMR=cardiac magnetic resonance, Echo=transthoracic echocardiogram, EF=ejection fraction, NYHA=New York Heart Association, RN=Radionuclide, SVR=surgical ventricular restoration
