# Supplementary material for: Perioperative changes in left ventricular systolic function following surgical revascularization
Source: PLoS One. 2022 Nov 10;17(11):e0277454. doi: 10.1371/journal.pone.0277454 (PMC9648779; doi:10.1371/journal.pone.0277454)
Supplement: S2 Table — (DOCX) [file pone.0277454.s002.docx]

**Supplemental table 2. Pre- and post-CABG LVEF by imaging modality**

|  | ***Preoperative LVEF****^* | ***Postoperative LVEF****^* | ***Difference in LVEF****^* | ***P value*** |
| --- | --- | --- | --- | --- |
| Echo (*n* = 191) | 29.7 (8.1) | 33.1 (10.1) | 3.4 (10.3) | <0.0001* |
| CMR (*n* = 273) | 27.4 (10.7) | 32.6 (13.4) | 5.2 (12.1) | <0.0001* |
| RN (*n* = 85) | 27.9 (7.7) | 33.8 (9.9) | 6.0 (8.1) | <0.0001* |

Abbreviations: CABG=coronary artery bypass graft, CMR=cardiac magnetic resonance, Echo=transthoracic echocardiogram, LVEF=left ventricular ejection fraction, RN=Radionuclide

^Mean (SD)

*Paired t-test
